# Genotypic selection and trait variation in sweet orange (*Citrus sinensis* L. Osbeck) dataset of Bangladesh

**DOI:** 10.1016/j.dib.2024.110333

**Published:** 2024-03-16

**Authors:** Mohammad Anwar Hossain Khan, M.A. Rahim, Mahbub Robbani, Fakhrul Hasan, Md. Rezwan Molla, Sanjida Akter, Abul Fazal Mohammad Shamim Ahsan, Zakaria Alam

**Affiliations:** a Tuber Crops Research Centre, Bangladesh Agricultural Research Institute, Gazipur 1701, Bangladesh; b Department of Horticulture, Bangladesh Agricultural University, Bangladesh; c Patuakhali Science and Technology University, Bangladesh; d Bangabandhu Sheikh Mujibur Rahman Agricultural University, Bangladesh; e Bangladesh Agricultural Research Council, Bangladesh; f Entomology Division, Bangladesh Rice Research Institute, Gazipur, Bangladesh; g Physiology Division, Bangladesh Agricultural Research Institute, Gazipur 1701, Bangladesh

**Keywords:** Citrus, BLUPs, Selection accuracy, Genetic variance, Broad sense heritability, Selection gain, MGIDI

## Abstract

The dataset primarily focused on selecting genotypes of sweet oranges based on their phenotypic performances. The dataset resulted significant variations in the best linear unbiased predictions (BLUPs) of 20 out of 21 traits, including leaves, flowers, fruits, and seeds. A strong positive correlation (r= 0.73 to 0.95) was observed among the majority of morphological traits. The sweet orange genotypes demonstrated considerable genetic variance, surpassing 65% for almost all traits, with a selection accuracy exceeding 92%. Using the multi-trait genotype-ideotype distance index (MGIDI), CS Jain-001 emerged as the top-ranked genotype, followed by BAU Malta-3 and CS Jain-002 in order of desirability. The broad sense heritability of selected traits was above 75.60%, and the selection gain reached a maximum of 12.60. These identified genotypes show promise as potential parent donors in breeding programs, leveraging their strengths and weaknesses to develop promising varieties in Bangladesh.

Specifications Table

**Table:** 

Subject	Agricultural and Biological Science
Specific subject area	Agronomy and Crop Science
Data format	Raw
Type of data	Table and Figures
How the data were collected	The morphological traits of sweet oranges were recorded following the guidelines outlined in the International Plant Genetic Resources Institute (IPGRI) descriptor of citrus
Data source location	The present investigation was conducted at Citrus Research Station (CRS), situated in Jaintiapur, Sylhet (24.8949° N and 91.8687° E). The experimental site falls under the classification of AEZ-20 (Eastern Surmakusiyara flood plains), which encompasses a diverse range of geographical features, including alluvial fans, back swamps, flood plains, flat hills, solitary hills, piedmont plains, point bars, and ridges.
Data accessibility	https://data.mendeley.com/datasets/g578j8tvsp/1

## 1 Value of the Data

• The dataset illustrates the connections among morphological traits, aiding in the identification of genotypes exhibiting substantial genetic variation. This offers breeders a broad genetic reservoir, allowing for the incorporation of unique and resilient characteristics into upcoming sweet orange varieties. This contributes to the sustainable development of sweet orange cultivation in the long run.
• The findings of the dataset equip farmers with the information necessary for making well-informed decisions regarding the cultivation of sweet orange genotypes. By pinpointing top-performing varieties through the MGIDI index, farmers can anticipate reliable and consistent performance. The focus on traits that significantly enhance selection accuracy (exceeding 92%) assists farmers in fine-tuning their cultivation methods to achieve improved yields and economic gains. This knowledge empowers farmers to choose varieties that match their specific requirements, promoting optimal resource utilization and enhancing overall farm profitability.
• The assessment of strengths and weaknesses in chosen genotypes is pivotal for advancing sustainable agricultural methods. By capitalizing on the strengths of specific genotypes, breeders can create varieties that cater to farmers' preferences, in harmony with the increasing global emphasis on sustainable agriculture.

## 2 Background

The sweet orange genotypes exhibit considerable variation in their morphologies, encompassing diverse attributes such as the size and shape of the canopy, the color, size, type, and season of ripening fruit, as well as the quantity of seeds per fruit [1]. Diversity in agricultural traits [2], [3], [4] offer an exceptional foundation for crop improvement. Skilled plant breeders often consider specific combinations of phenotypic characteristics from a diverse collection to develop new genotypes that exhibit exceptional performance. This concept, known as ideotype, was introduced by Donald [5] in wheat breeding. The core concept of ideotype design aims to enhance crop performance by selecting genotypes that exhibit multiple desirable traits simultaneously. The multi-trait genotype–ideotype distance index (MGIDI), a recently developed method, is created to aid in genotype selection based on breeding values, incorporating information from several traits [6,7]. The dataset contains four tables and three figures.

## 3 Data Description

**Fig. 1:**
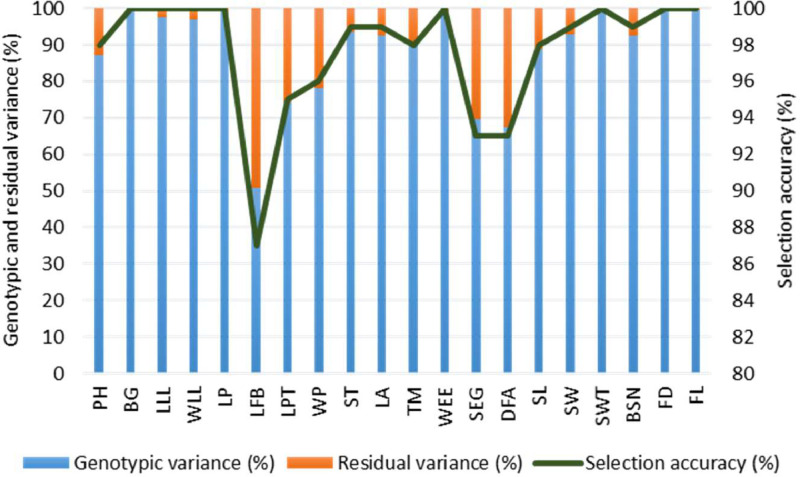
Genotypic variance (%), residual variance (%) and selection accuracy for 20 quantitative traits of 8 sweet orange genotypes obtained from restricted maximum likelihood (RELM) test. ^PH^ plant height, ^BG^ base grith, ^LLL^ length of leaf lamina, ^WLL^ width of leaf lamina, ^LP^ length of pedicel, ^LFB^ length of flower bud, ^LPT^ length of petal, ^WP^ width of petal, ^ST^ number of stamens, ^LA^ length of anther, ^TM^ thickness of mesocarp, ^WEE^ width of epicarp at equatorial area, ^SEG^ number of segments per fruit, ^DFA^ diameter of fruit axis, ^SL^ seed length, ^SW^ seed width, ^SWT^ single seed weight, ^BSN^ bold seed number per fruit, ^FD^ fruit diameter and ^FL^ fruit length.

In the restricted maximum likelihood model (RELM), the likelihood ratio test (LRT) indicated statistically significant differences at a 5% significance level among the best linear unbiased prediction (BLUP) values for all assessed traits, with the exception of AT, across the genotypes under investigation (Table 1). The genotypic variance components obtained from RELM suggest that genetic effects have a substantial impact, exceeding 65%, in comparison to residual variance for all traits except LFB (Fig. 1). Moreover, the selection accuracy exceeded 92% for all traits except LFB (Fig. 1).

**Table 1:** BLUP values for 20 quantitative traits of eight sweet orange genotypes obtained using RELM.

Genotypes	PH	BG	LLL	WLL	LP	LFB	LPT	WP	ST	LA	TM	WEE	SEG	DFA	SL	SW	SWT	BSN	FD	FL
BARIMalta-1	1.52	37.5	85	51	7.33	17.5	18.8	8	21	2.04	5.09	56	11.1	22.3	12.1	7.02	3.36	13.7	73.7	56.7
BAUMalta-1	1.04	42.2	91	53	7.33	16.8	17.9	8	20	2.11	7.02	61	11.1	20.3	13	6.69	3.76	13	68.2	66.7
BAUMalta-3	2.85	39.8	98.9	60.9	8.66	16	17.9	9.83	27.9	2.49	9.91	76	11.1	23.4	15.9	8.64	5.36	14	87.7	80
CSJain-001	2.66	46.7	95	53	8	15.5	18.8	9.23	27.2	2.49	8.95	76	12.6	22.9	14	8.32	3.7	12.7	92	82
CSJain-002	1.42	46.2	89.6	46	8.66	16	16.1	8.61	23	2.11	9.91	66	12.9	22	18.5	11.2	6.51	20.8	89	82
CSJain-003	1.33	36.2	81.1	45.1	8	14.8	14.6	6.48	18.1	2.52	7.98	56	14.6	22.9	11.4	6.05	2.33	11.4	65	60
CSRam-001	2.22	38.5	86	49	7.33	16	17	7.39	22	2.11	7.02	66	12.3	21.1	14	6.69	3.86	13	76.7	63
VariegatedMalta	1.99	41.9	86	46.3	7.33	16	16.1	6.78	18.1	2.49	3.79	46.1	11.1	20.8	12.7	8.32	4.16	16	63.7	62
Significance	*																			

⁎ Significant at 5% probability level in likelihood ratio test (LRT), ^PH^ plant height, ^BG^ base grith, ^LLL^ length of leaf lamina, ^WLL^ width of leaf lamina, ^LP^ length of pedicel, ^LFB^ length of flower bud, ^LPT^ length of petal, ^WP^ width of petal, ^ST^ number of stamens, ^LA^ length of anther, ^TM^ thickness of mesocarp, ^WEE^ width of epicarp at equatorial area, ^SEG^ number of segments per fruit, ^DFA^ diameter of fruit axis, ^SL^ seed length, ^SW^ seed width, ^SWT^ single seed weight, ^BSN^ bold seed number per fruit, ^FD^ fruit diameter and ^FL^ fruit length.

**Fig. 2:**
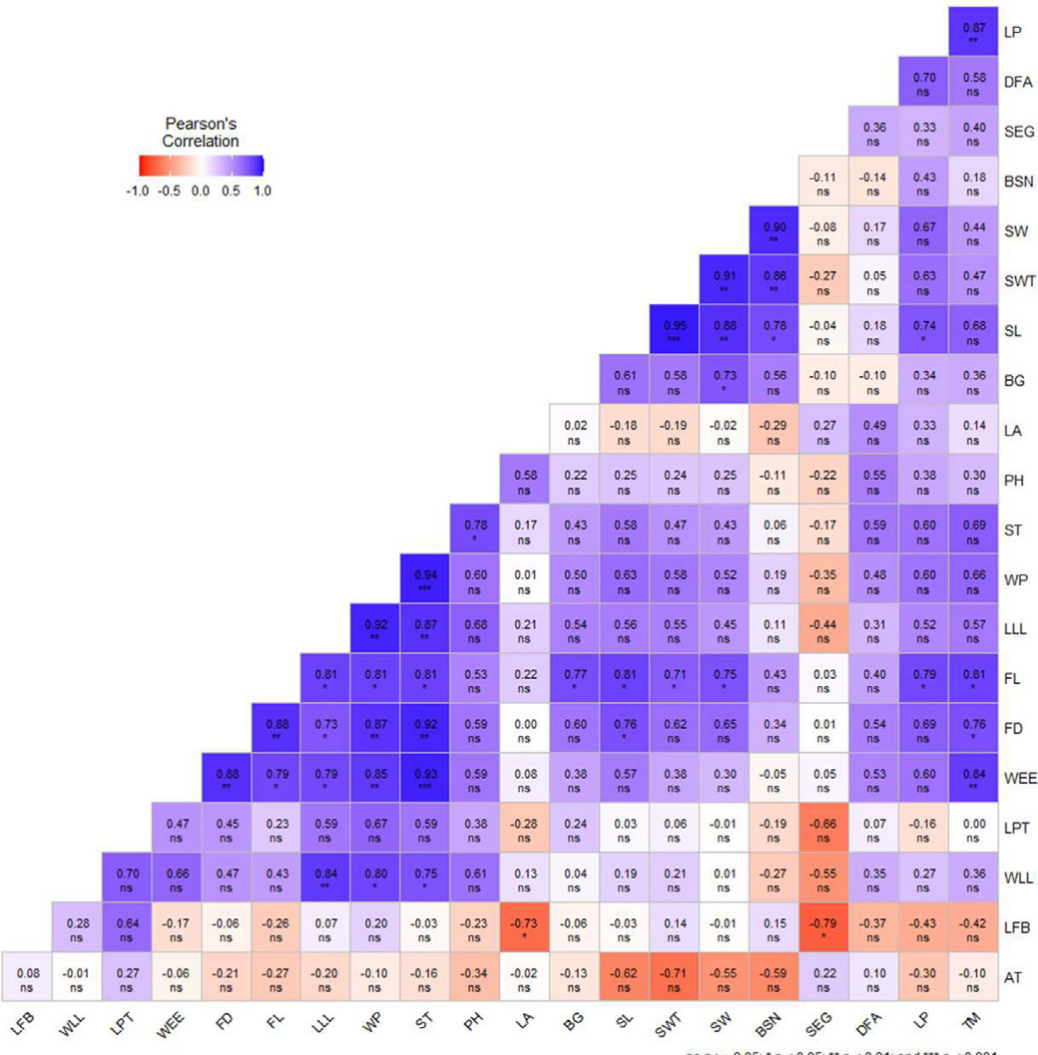
Coefficient of correlation matrix (Pearson's correlation) of 21 traits of eight sweet orange genotypes. ^AT^ age of tree, ^PH^ plant height, ^BG^ base grith, ^LLL^ length of leaf lamina, ^WLL^ width of leaf lamina, ^LP^ length of pedicel, ^LFB^ length of flower bud, ^LPT^ length of petal, ^WP^ width of petal, ^ST^ number of stamens, ^LA^ length of anther, ^TM^ thickness of mesocarp, ^WEE^ width of epicarp at equatorial area, ^SEG^ number of segments per fruit, ^DFA^ diameter of fruit axis, ^SL^ seed length, ^SW^ seed width, ^SWT^ single seed weight, ^BSN^ bold seed number per fruit, ^FD^ fruit diameter and ^FL^ fruit length.

A significant (p<0.05) correlation coefficient (r= 0.73 to 0.95) was identified among the morphological traits of sweet orange, as illustrated in Fig. 2. The findings indicated noteworthy positive correlations between various traits such as LP and TM, FL and TM, FD and TM, WEE and TM, SL and LP, FL and LP, SW and BSN, SWT and BSN, SL and BSN, SWT and SW, SL and SW, FL and SW, SL and SWT, FL and SWT, FL and SL, FD and SL, FL and BG, ST and PH, WP and ST, LLL and ST, FL and ST, FD and ST, WEE and ST, WLL and ST, LLL and WP, FL and WP, FD and WP, WEE and WP, WLL and WP, FL and LLL, FD and LLL, WEE and LLL, FD and FL, WEE and FL, WEE and FD. Additionally, a significant negative correlation (r=0.73) was observed between SEG and LFB, as well as LA and LFB.

**Fig. 3:**
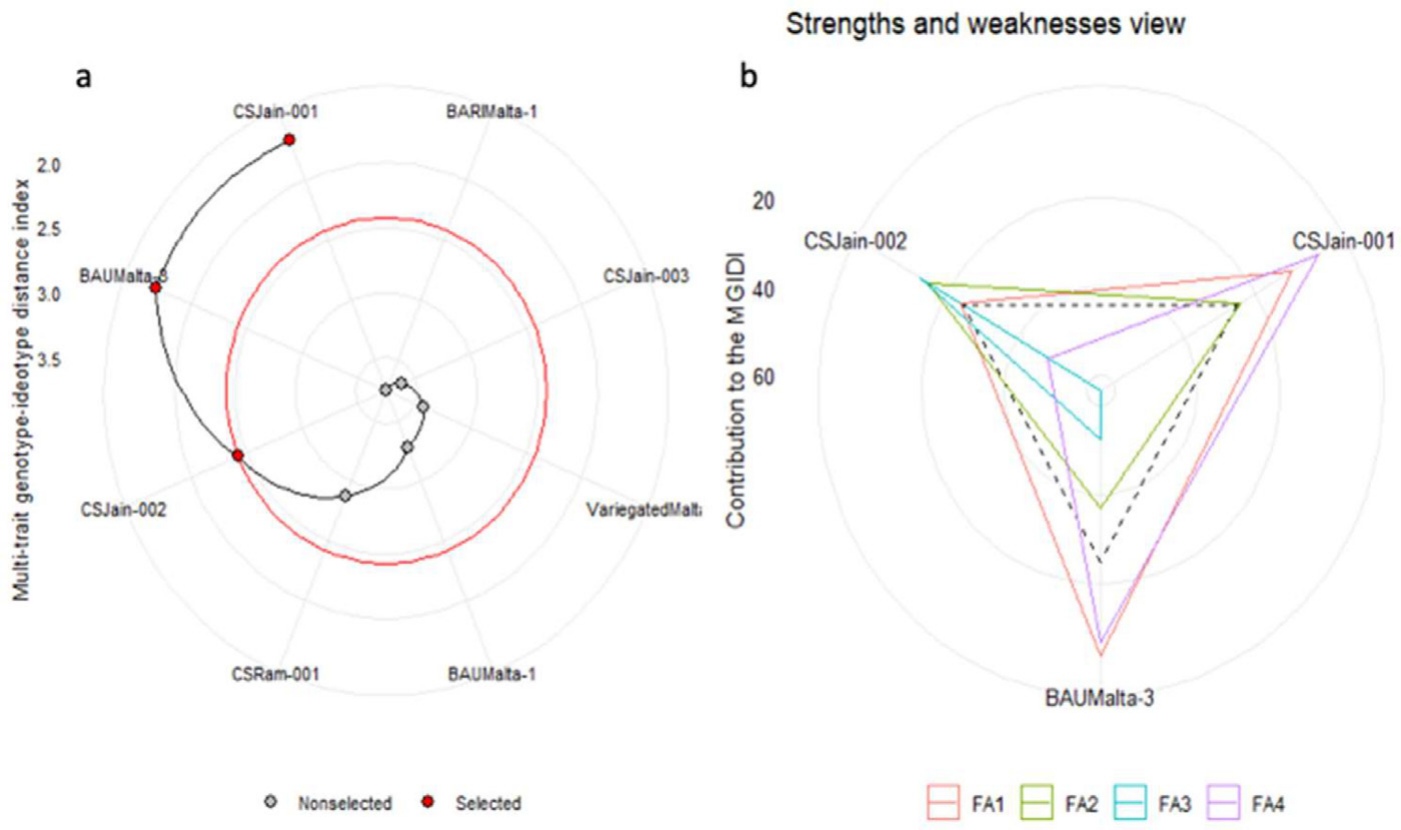
Selection of sweet orange genotypes through MGIDI index (a) and the strengths and weaknesses of selected genotypes (b).

In Fig. 3a, the arrangement of eight sweet orange genotypes according to the multi-trait genotype-ideotype index (MGIDI) is depicted. The genotypes are organized in a descending order of MGIDI index values, where the highest value is positioned at the center and the lowest at the outer circle. The red circle in Fig. 3a represents a selection intensity (SI) of 40%, indicating an increasing selection sense for all traits except LFB, as determined by the MGIDI selection index. The successful attainment of the selection sense goal is highlighted in Table 2. The red dots within the circle represent the sweet orange genotypes selected through the MGIDI index (Fig. 3a). The selection gains (SG) ranged from 0.07 to 12.6, and heritability (*h^2^*) ranged from 75.60% to 100% (Table 2). Notably, CS Jain-001 was identified as the most desirable genotype, followed by BAU Malta-3 and CS Jain-002, according to the MGIDI index (Fig. 3a). Fig. 3b illustrates the strengths and weaknesses of these selected genotypes using four identified factors (FA) (Table 2) and their loadings (Table 4) derived from significant principal components (Table 3). The arrangement of factors concerning genotypes in Fig. 3b signifies their impact, with dotted lines indicating the average performance in factor contribution. Higher factor values moving toward the center indicate weaknesses, while lower values denote strengths (Fig. 3b). Within Fig. 3b, Factor 1 (FA1) incorporates traits such as LLL, LP, WP, ST, TM, WEE, DFA, FD, and FL; Factor 2 (FA2) includes WLL, LFB, LPT, and SEG; Factor 3 (FA3) comprises BG, SL, SW, SWT, and BSN; and Factor 4 (FA4) encompasses PH and LA, as outlined in Table 2. The positions of the selected genotypes in Fig. 3b reflect their strengths and weaknesses in relation to these factors.

**Table 2:** Factor contribution (FA), broad-sense heritability (*h^2^*) and selection gain (SG) obtained using MGIDI selection index for 20 quantitative traits of three selected genotypes of sweet orange

Quantitative traits	Factor	$\bar{X}$_o_	$\bar{X}$_s_	*h^2^*	SG	Sense	Goal
LLL	FA1	89.1	94.5	99.20	5.37	Increase	100
LP	FA1	7.83	8.44	99.70	0.61	Increase	100
WP	FA1	8.04	9.22	91.50	1.08	Increase	100
ST	FA1	22.2	26	97.80	3.77	Increase	100
TM	FA1	7.46	9.59	96.50	2.06	Increase	100
WEE	FA1	62.9	72.6	99.70	9.73	Increase	100
DFA	FA1	22	22.8	86.00	0.69	Increase	100
FD	FA1	77	89.5	99.90	12.60	Increase	100
FL	FA1	69	81.3	99.80	12.20	Increase	100
WLL	FA2	50.5	53.3	99.00	2.74	Increase	100
LFB	FA2	16.1	15.9	75.60	-0.18	Decrease	100
LPT	FA2	17.2	17.6	89.70	0.40	Increase	100
SEG	FA2	12.1	12.2	87.30	0.07	Increase	100
BG	FA3	41.1	44.2	100	3.11	Increase	100
SL	FA3	14	16.1	95.90	2.08	Increase	100
SW	FA3	7.87	9.4	97.50	1.49	Increase	100
SWT	FA3	4.13	5.19	100	1.06	Increase	100
BSN	FA3	14.3	15.8	97.40	1.48	Increase	100
PH	FA4	1.88	2.31	95.30	0.41	Increase	100
LA	FA4	2.3	2.36	97.40	0.07	Increase	100

$\bar{X}$_o_= observed mean, $\bar{X}$_s_=predicted mean obtained from BLUP values, ^PH^ plant height, ^BG^ base grith, ^LLL^ length of leaf lamina, ^WLL^ width of leaf lamina, ^LP^ length of pedicel, ^LFB^ length of flower bud, ^LPT^ length of petal, ^WP^ width of petal, ^ST^ number of stamens, ^LA^ length of anther, ^TM^ thickness of mesocarp, ^WEE^ width of epicarp at equatorial area, ^SEG^ number of segment per fruit, ^DFA^ diameter of fruit axis, ^SL^ seed length, ^SW^ seed width, ^SWT^ single seed weight, ^BSN^ bold seed number per fruit, ^FD^ fruit diameter and ^FL^ fruit length.

**Table 3:** Principal components (PCs), eigenvalues, variance (%) of PCs and cumulative variance (%) of all PCs obtained from factor analysis of MGIDI index

PC	Eigenvalues	Variance (%) of principal components	Cumulative variance (%)
PC1	9.64	48.2	48.2
PC2	3.7	18.5	66.7
PC3	3.5	17.5	84.3
PC4	1.22	6.12	90.4
Noise	1.93	9.63	-

**Table 4:** Factor loadings (FA), communality and uniquenesses of 20 quantitative traits of sweet orange genotypes

Traits	FA1	FA2	FA3	FA4	Communality	Uniquenesses
PH	-0.48	0.32	0.06	-0.67	0.79	0.21
BG	-0.17	0.15	0.76	-0.15	0.64	0.36
LLL	-0.65	0.52	0.36	-0.32	0.93	0.07
WLL	-0.63	0.66	-0.1	-0.2	0.88	0.12
LP	-0.68	-0.36	0.51	-0.18	0.89	0.11
LFB	-0.19	-0.76	-0.03	-0.57	0.94	0.06
LPT	-0.39	0.85	-0.1	0.12	0.9	0.1
WP	-0.79	0.46	0.36	-0.07	0.98	0.02
ST	-0.86	0.34	0.25	-0.23	0.98	0.02
LA	-0.11	-0.26	-0.11	-0.93	0.96	0.04
TM	-0.87	-0.31	0.31	0	0.94	0.06
WEE	-0.94	0.15	0.15	-0.08	0.93	0.07
SEG	-0.19	-0.94	-0.15	-0.01	0.95	0.05
DFA	-0.73	-0.26	-0.11	-0.3	0.7	0.3
SL	-0.46	-0.03	0.85	0.12	0.95	0.05
SW	-0.21	-0.02	0.96	-0.05	0.96	0.04
SWT	-0.26	0.14	0.91	0.08	0.92	0.08
BSN	0.13	-0.09	0.94	0.21	0.96	0.04
FD	-0.81	0.14	0.48	-0.04	0.91	0.09
FL	-0.68	0.04	0.65	-0.25	0.95	0.05

^PH^ plant height, ^BG^ base grith, ^LLL^ length of leaf lamina, ^WLL^ width of leaf lamina, ^LP^ length of pedicel, ^LFB^ length of flower bud, ^LPT^ length of petal, ^WP^ width of petal, ^ST^ number of stamens, ^LA^ length of anther, ^TM^ thickness of mesocarp, ^WEE^ width of epicarp at equatorial area, ^SEG^ number of segments per fruit, ^DFA^ diameter of fruit axis, ^SL^ seed length, ^SW^ seed width, ^SWT^ single seed weight, ^BSN^ bold seed number per fruit, ^FD^ fruit diameter and ^FL^ fruit length.

## 4 Experimental Design, Materials and Methods

### 4.1 Description of Experimental Site

The present investigation was conducted at Citrus Research Station (CRS), situated in Jaintiapur, Sylhet (24.8949° N and 91.8687° E), over the course of the period spanning from February 2020 to December 2021 (one growing season). The experimental site falls under the classification of AEZ-20 (Eastern Surmakusiyara flood plains), which encompasses a diverse range of geographical features, including alluvial fans, back swamps, flood plains, flat hills, solitary hills, piedmont plains, point bars, and ridges. The soil texture may exhibit variation from clay loam to sandy loam, with certain regions predominantly composed of sand and the subsoil being constituted by a higher proportion of clay [8]. Furthermore, in AEZ-20, the soil organic matter content is generally elevated, while the pH remains neutral [9]. The location of sweet orange orchard is situated within a subtropical climatic region, characterized by elevated levels of precipitation due to the influence of monsoon air originating from the south-west.

### 4.2 Plant Materials

In this particular study, a collection of eight sweet orange genotypes, namely CS Jain-001, CS Jain-002, CS Jain-003, BAU Malta-3, BAU Malta-1, BARI Malta-1, CS Ram-001, and Variegated Malta, were employed. The aforementioned genotypes were cultivated within the orchards of experimental sites established in Sylhet, Bangladesh.

### 4.3 Design of Experiment and Data Collection

The experiment comprised of three replications that were completely randomized, with each replication consisting of ten trees per genotype. A total of 240 trees, aged between 7 and 8 years, were included within the radius of the characterization study. The spacing between trees and rows was upheld at 4 meters and 3 meters, respectively. The sweet orange genotypes were cultivated using pummelo rootstock, and no incidence of insect or pest infestation was observed during the course of the investigation. The morphological traits of sweet oranges were recorded following the guidelines outlined in the International Plant Genetic Resources Institute (IPGRI) descriptor of citrus [10]. The traits were age of tree (AT), plant height (PH), base grith (BG), length of leaf lamina (LLL), width of leaf lamina (WLL), length of pedicel (LP), length of flower bud (LFB), length of petal (LPT), width of petal (WP), number of stamens (ST), length of anther (LA), thickness of mesocarp (TM), width of epicarp at equatorial area (WEE), number of segments per fruit (SEG), diameter of fruit axis (DFA),seed length (SL), seed width (SW), single seed weight (SWT), bold seed number per fruit (BSN), fruit diameter (FD) and fruit length (FL). The measurements were taken from three trees in each replication, and the results were averaged.

### 4.4 Statistical Analysis

We conducted Pearson's correlation analysis on genotypic mean performances, examining 21 morphological traits. The indices for calculating the genetic parameters and breeding values (Table 5) using multi-trait genotype-ideotype distance index (MGIDI) for ranking sweet orange genotypes were analysed using “metan” package of R software [11]. The calculation of the MGIDI index aimed to identify the top-ranked genotype (Eq. i). Prior to making selections, ideotype design and trait rescaling were undertaken (Eq. ii). For assessing the significance of each trait concerning genotypes, a linear mixed model (Eq. iii) was employed. Calculations of the observed mean (${\bar{X}}_{o}$) and best linear unbiased prediction (BLUP) values for the predicted mean (${\bar{X}}_{s}$) of genotypes were performed to determine selection gain (Eq. iv). Variance components obtained from MGIDI analysis were used to compute broad-sense heritability (h^2^) based on the mean performance of genotypes (Eq. v). Factor analysis (Eq. vi) and factor loadings (vii) with varimax rotation criteria [12] were executed with an aim of increase selection for all traits, except LFB, with a 40% selection intensity. Additionally, the strengths and weaknesses of selected genotypes (viii) were identified using factor loadings. The genetic parameters, including genotypic variance, residual variance, and selection accuracy, were calculated using the mixed-effect model (ix) within a randomized complete block design. In this model, replicates were treated as fixed, while genotypes were considered random.

**Table 5:** The equations for calculating the genetic parameter and breeding values using MGIDI to select superior sweet orange genotypes.

Selection indices	Equation No.	Reference
Multi trait genotype-ideotype index, $MGIDI_{i}={\left[\sum_{j=1}^{f}{\left(\gamma_{ij}-\gamma_{j}\right)}^{2}\right]}^{0.5}$	i	[6]
Ideotype design and rescaling of traits, $rX_{ij}=\frac{\eta_{nj}-\varphi_{nj}}{\eta_{oj}-\varphi_{oj}}\times \left(\theta_{ij}-\eta_{0j}\right)+\eta_{nj}$	ii	
Linear mixed model, y=Xb+Zu+e	iii	
Selection gain, $SG\left(\%\right)=\frac{\left({\bar{X}}_{s}-{\bar{X}}_{o}\right)\times h^{2}}{{\bar{X}}_{o}}\times 100$	iv	
Broad sense heritability, $h^{2}={\overset{⌢}{\sigma }}_{\alpha }^{2}/\left({\overset{⌢}{\sigma }}_{\alpha }^{2}+{\overset{⌢}{\sigma }}_{\alpha }^{2}/r\right)$	v	
Factor analysis, X=μ+Lf+ε	vi	
Factor loadings, $F=Z{\left(A^{T}R^{-1}\right)}^{T}$	vii	
Strength and weaknesses of selected genotypes, $\omega_{ij}=\frac{\sqrt{D_{ij}^{2}}}{\sum_{j=1}^{f}\sqrt{D_{ij}^{2}}}$	viii	
Mixed-effect model, $y_{ij}=m+g_{i}+r_{j}+e_{ij}$	ix	[13]

## Limitations

This dataset, although instrumental for the selection of sweet orange genotypes based on phenotypic characteristics, is constrained by the absence of molecular or genomic information associated with these genotypes. This limitation impedes a comprehensive elucidation of the molecular mechanisms underpinning the observed phenotypic traits. The integration of genomic data has the potential to augment the dataset's analytical depth, facilitating a more exhaustive investigation into the genetic foundations of these traits and enhancing the precision of genotype selection methodologies within breeding programs.

## Ethics Statement

All authors have read and follow the ethical requirements for publication in Data in Brief and our work meets these requirements. Our work does not involve studies with animals and humans.

## CRediT authorship contribution statement

**Mohammad Anwar Hossain Khan:** Conceptualization, Investigation. **M.A. Rahim:** Supervision, Methodology. **Mahbub Robbani:** Supervision, Methodology. **Fakhrul Hasan:** Supervision, Methodology. **Md. Rezwan Molla:** Data curation, Validation. **Sanjida Akter:** Data curation, Validation. **Abul Fazal Mohammad Shamim Ahsan:** Data curation, Validation. **Zakaria Alam:** Visualization, Software, Writing – original draft, Writing – review & editing.

## Data Availability

Genotypic selection and trait variation in sweet orange (Citrus sinensis L. Osbeck) dataset of Bangladesh (Original data) (Mendeley Data)
